# Low-Level Detection of Poly(amidoamine) PAMAM Dendrimers Using Immunoimaging Scanning Probe Microscopy

**DOI:** 10.1155/2012/341260

**Published:** 2012-02-08

**Authors:** Chevelle A. Cason, Thomas A. Fabré, Andrew Buhrlage, Kristi L. Haik, Heather A. Bullen

**Affiliations:** ^1^Department of Chemistry, Northern Kentucky University, Highland Heights, KY 41099, USA; ^2^Department of Biological Sciences, Northern Kentucky University, Highland Heights, KY 41099, USA

## Abstract

Immunoimaging scanning probe microscopy was utilized for the low-level detection and quantification of biotinylated G4 poly(amidoamine) PAMAM dendrimers. Results were compared to those of high-performance liquid chromatography (HPLC) and found to provide a vastly improved analytical method for the low-level detection of dendrimers, improving the limit of detection by a factor of 1000 (LOD = 2.5 × 10^−13^ moles). The biorecognition method is reproducible and shows high specificity and good accuracy. In addition, the capture assay platform shows a promising approach to patterning dendrimers for nanotechnology applications.

## 1. Introduction

Dendrimers are at the forefront of research in nanoscience due to the many interesting properties of these macromolecular systems including their precise architecture, highly reproducible shape, high uniformity and purity, low immunogenicity and toxicity, high loading capacity, and high shear resistance [1–5]. They have shown a great deal of versatility with applications in numerous areas such as drug delivery [6, 7], gene therapy [8, 9], chemotherapy [10], electrochemistry [11, 12], metal recovery [13], catalysis [14, 15], and sensors [16–18]. Development of new low-level detection and quantification methods is needed with the utilization of these nanomaterials. Currently, high-performance liquid chromatography (HPLC) is the predominate approach reported for dendrimer quantification [19, 20]. However, the primary focus of HPLC, along with capillary electrophoresis, has been to evaluate dendrimer purity and degree of conjugation [21–27]. Little has been reported within the literature with regard to the advancement of new quantification methods for dendrimers. 

This work introduces a biorecognition readout technique that has the potential to provide low-level detection of dendrimers. Biotinylated poly(amidoamine) PAMAM dendrimers were chosen as a model target. PAMAM dendrimers, which are highly water soluble, represent the most widely studied class of dendrimers. Functionalization of PAMAM dendrimer surfaces has proven useful in their utilization for various applications including drug delivery and chemical sensing [5, 6, 16]. Biotin-labeled dendrimers have been utilized in tumor [28] and antibody [29] targeting studies and biosensor design [30]. Biotinylated PAMAM dendrimers may also have the potential for delivering therapeutic drugs to the brain [31, 32].

We report here a readout method using an immunoassay platform and scanning probe microscopy (SPM) for low-level quantification of biotinylated G4 PAMAM dendrimers. The assay takes advantage of the documented specificity of biotin-avidin. Results are correlated with HPLC analysis. In addition, we briefly highlight the potential of this capture assay platform to selectively pattern PAMAM dendrimers onto a surface. Patterning of nanoparticles is relevant to a wide variety of applications in the fields of sensing, drug delivery, or development of nanodevices [33–35]. Dendritic architectures show promise in designing and developing sensor platforms with high sensitivity and stability [16].

## 2. Experimental

### 2.1. Reagents

Poly(amidoamine) PAMAM dendrimers [core: ethylene diamine] (G = 4) *dendri-*PAMAM-(NH_2_)_32 _ were obtained from Dendritic Nanotechnologies, Inc. (Mt. Pleasant, MI). Biotinylated PAMAM dendrimers were prepared using sulfo-NHS-LC-biotin (Pierce EZ-Link Kit) as described previously [36]. Briefly, a 3 : 1 molar ratio of biotin/PAMAM dendrimers in 0.1 M phosphate buffer saline (PBS) was allowed to react for 2 h on an orbital shaker. Excess, unreacted biotin was then removed using Microcon filters (Millipore. Bedford, MA, USA). The biotinylation of dendrimers was evaluated using NMR spectroscopy. Biotinylated dendrimers were resuspended (1.0 mg/mL) in 1.0 M PBS until used. Octadecanethiol (ODT), 3,3′-dithio-bis(propionic acid N-hydroxysuccinimide ester) (DSP), bovine serum albumin (BSA), Triton X-100, and avidin >98% were obtained from Sigma (Sigma-Aldrich, St. Louis, MO). Avidin conjugated to Alexa Fluor 488 was purchased from Invitrogen (Invitrogen, Carlsbad, CA). Poly(dimethyl siloxane) (PDMS) was obtained from Dow Corning (Midland, MI). All organic solvents used were analytical, HPLC grade, from Sigma (Sigma-Aldrich, St. Louis, MO). DI water was obtained using a Milli-Q plus water purification system (Millipore, Bedford, MA). PBS and Borate buffers were prepared from Pierce buffer packs (Pierce Protein Research Products, Rockford, IL).

### 2.2. Capture Substrate Preparation

**Figure 1:**
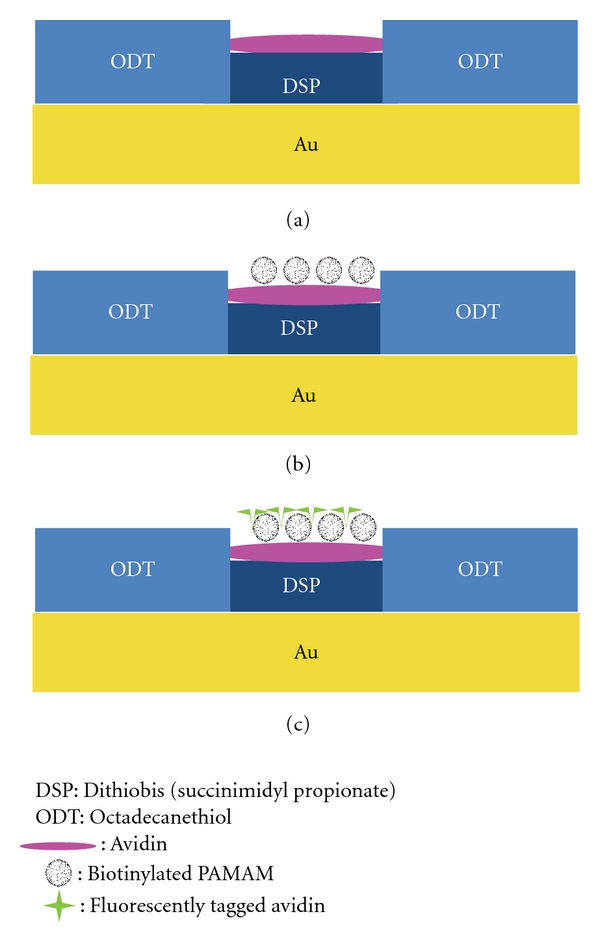
Schematic of capture assay platform: (a) immobilize avidin onto Au substrate with controlled surface area using DSP and (b) expose substrate to biotinylated PAMAM dendrimers. (c) The captured biotinylated PAMAM dendrimers can be fluorescently labeled for additional verification of immobilization and patterning of dendrimers.

A modified approach was used for preparation of the capture substrate [37–39] utilizing template-stripped gold (TSG) for SPM imaging, as shown in Figure 1. TSG was prepared by evaporating gold onto p-type silicon wafers (University Wafer) with a resistive evaporator and affixing 1 × 1 cm glass pieces (ultrasonically cleaned 30 min each in diluted Contrad 70, DI water, and methanol) using two-part epoxy (Epoxy Technology) followed by curing at 150°C for 2 h. The glass pieces were gently detached from the silicon wafer revealing a smooth gold surface atop the glass chip.

The TSG substrates were exposed for ~30 s to an ODT soaked PDMS stamp (with a 3 mm diameter hole cut in the center), rinsed with ethanol, and dried under high-purity nitrogen. The substrates were then placed in a 0.1 mM solution of DSP in ethanol overnight. The capture platform was then rinsed with ethanol and dried under N_2_. This formed the DSP-based adlayer in the areas on the substrate not covered by ODT. The hydrophobic ODT localized reagents in a confined sample area (3 mm ODT spot size) for the capture assay platform.

To form the capture avidin surface, a 20 *μ*L aliquot of avidin solution (500 *μ*g/mL diluted in 50 mM borate buffer) was placed on top of the sample area and allowed to incubate for 6 h at room temperature in a humidity chamber. Substrates were then rinsed with 5 mL of 10 mM PBS (with 0.1% Triton X-100), and the surface area was incubated with a 20 *μ*L solution of blocking buffer (1% (w/v) BSA in 20 mM borate buffer with 0.1% Triton X) for 5 h, followed by rinsing with 5 mL of 10 mM PBS. The capture substrate was then exposed to 20 *μ*L aliquots of various concentrations of biotinylated G4 PAMAM dendrimers diluted in 10 mM PBS for 8 h at room temperature in a humidity chamber. Substrates were then rinsed with 5 mL of 10 mM borate buffer before drying with nitrogen. A blank sample consisted of all stages except the avidin capture surface. 

To demonstrate patterning of biotinylated G4 PAMAM dendrimers, a PDMS stamp with a positive structure of 10 *μ*m wide stripes separated by 5 *μ*m was utilized. Preparation of the patterned substrate consisted of all stages, with the following modifications: (1) a sample volume of 200 *μ*L was used instead of 20 *μ*L and (2) after incubation with biotinylated G4 PAMAM dendrimers, the substrate was rinsed with 10 mM PBS and the dendrimers were fluorescently tagged by incubating the substrate with 200 *μ*L of 250 ug/mL of Alexa Fluor conjugated avidin. Following this step the substrates were rinsed with 5 mL of 10 mM borate buffer and dried with nitrogen.

### 2.3. Scanning Probe Microscopy

A Dimension 3100 Digital Instruments SPM was utilized in tapping mode equipped with 125 *μ*m n-doped silicon cantilevers with resonance frequencies between 110 and 220 kHz and typical scan rates of 0.75–1 Hz (NSC 14, MikroMasch). Typical scan rates were 1 Hz, and all images were acquired in air. For quantification, multiple areas of the capture surface were analyzed and the dendrimers in each 1 × 1 *μ*m image were enumerated manually.

### 2.4. High-Performance Liquid Chromatography

HPLC analysis of G4 biotinylated PAMAM dendrimers was conducted using parameters reported previously [36]. Briefly, a reversed phase HPLC system consisting of a Waters Breeze HPLC (Waters Corporation, Milford, MA, USA), equipped with a 717plus autosampler, 2487 dual *λ* UV detector, 5 *μ*m Symmetry300 C18 column (4.6 mm × 150 mm), and a Waters Sentry Symmetry C18 guard column was used. The mobile phase consisted of a linear gradient beginning with 90% water and 10% ACN (each with 0.14% TFA) at a flow rate of 1 mL/min reaching 68% water and 32% ACN over 10 minutes. The injection volume was 100 *μ*L, and detection of eluted samples was performed at 214 nm.

### 2.5. Fluorescent Microscopy

A Nikon E600 fluorescent microscope with a Mercury-100 W light source was utilized to take images at 100x total magnification.

## 3. Results and Discussion

**Figure 2:**
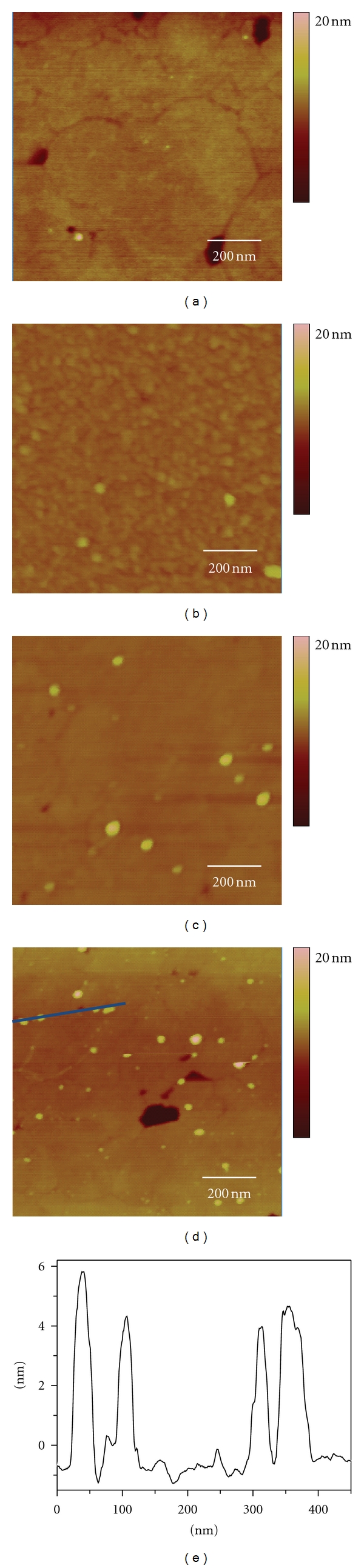
Representative SPM images of capture surface (1 × 1 *μ*m) of different concentrations of bound biotinylated G4 PAMAM dendrimers to avidin capture substrate: (a) blank and (b) 7.0 × 10^−3^ *μ*mol/L, (c) 7.0 × 10^−2^ *μ*mol/L, and (d) 3.28 × 10^−1^ *μ*mol/L; (e) a cross-sectional analysis of captured biotinylated G4 PAMAM dendrimers in (d).

The principle goal of this work was the development and application of capture platform for quantification of PAMAM dendrimers. A model assay was evaluated using biotinylated G4 PAMAM dendrimers and avidin. Avidin was immobilized onto a patterned thiolate layer, DSP, via succinimidyl ester chemistry (Figure 1). In the presence of biotinylated G4 PAMAM dendrimers, the immobilized avidin layer extracts the dendrimers. SPM analysis of the capture platform revealed the presence of nanometrically sized objects, ~4.5 nm in dimension, according to cross-sectional height profiles. These are consistent in size and shape of G4 biotinylated PAMAM dendrimers, taking into account tip convolution effects (Figure 2). At this scale, the tip convolution effects distorted the resolution in the lateral dimensions, as the dimensions of the particles are smaller than the radius of curvature of the AFM probe used (radius = 20 nm) [40, 41]. The dendrimers largely appear to be captured individually on the platform surface. In some cases dendrimers were found grouped together (2-3 dendrimer clusters) but could be individually distinguished using cross-sectional analysis. SPM analysis reveals that the dendrimers do not have a tendency to cluster together in domains, indicating that sedimentation is not a factor during the incubation. Analysis of control (blank) substrates, devoid of the avidin-specific capture surface, shows limited nonspecific binding (0.67 ± 0.45 dendrimers/*μ*m^2^) and that blocking steps are effective. 

**Figure 3:**
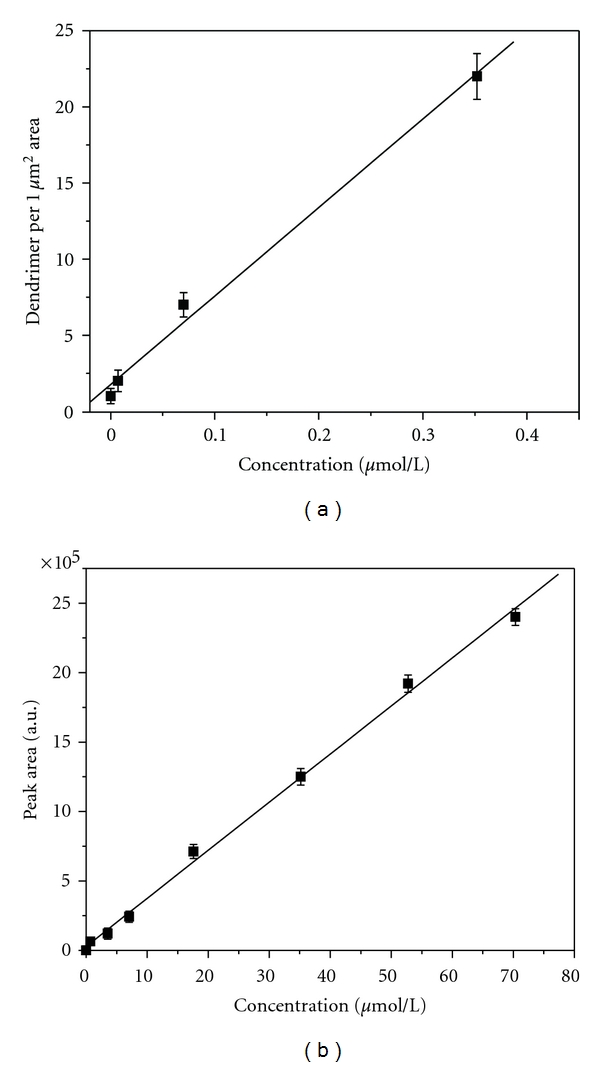
Calibration curves for (a) immunoimaging SPM assay and (b) HPLC analysis of G4 biotinylated PAMAM dendrimers. The SPM analysis represents the average number of dendrimers bound within a 1 × 1 *μ*m area for different concentrations of dendrimers. Data acquired from 10 capture substrates, with 10 images per sample (*y* = 58.1*x* + 1.76, *n* = 20, *r* ^2^ = 0.9964). The HPLC analysis represents an average of 10 samples for each concentration evaluated (*y* = 34620.85*x* + 28845.984, *n* = 10, *r* ^2^ = 0.9986).

The patterning of DSP provides a reproducible surface area for quantification. Figure 2 shows that the number of captured G4 biotinylated PAMAM dendrimers tracks with the increase in dendrimer solution concentration. The degree of bound dendrimers was similar for various different areas of the capture surface. A calibration plot of the amount of captured G4 biotinylated PAMAM dendrimers (determined by SPM analysis) as a function of solution concentration is shown in Figure 3(a). The number of captured dendrimers correlates with G4 biotinylated PAMAM dendrimer concentrations, showing a linear dynamic range under the concentrations range evaluated (7.0 × 10^−3^–3.28 × 10^−1^ *μ*mol/L). This readout method has the potential for low limits of detection. The limit of detection can be estimated as the signal three times above the measurement noise, where the noise is the sample blank response. This corresponds to a limit of detection of ~2.5×10^−13^ moles. Results were correlated with a standard HPLC analysis approach for quantification of dendrimers, as shown in Figure 3(b). HPLC quantification of dendrimers shows a linear dynamic range under the concentrations evaluated (7.0 × 10^−1^–70.35 *μ*mol/L) with a corresponding limit of detection of 1.8 × 10^−10^ moles. The capture assay platform improves the limit of detection by a factor of 1000. It should also be noted that the concentrations investigated by the capture assay were not detectable by HPLC analysis.

The capture assay platform has shown the potential for low-level detection and quantification of G4 biotinylated PAMAM dendrimers. Further improvements in the performance of this immunosorbent assay approach such as the reduction of incubation times may be possible by utilizing a rotating capture substrate to increase the flux of analyte to the capture surface [38]. In addition, the limit of detection may be further improved by reducing the area dimension of the DSP capture platform. The range of dendrimers that can be quantified using this approach is potentially limited to the analyte capture surface and labeling strategy of the dendrimers. This quantification method can be applied to any type of immunosorbant assay approach (e.g., antibody antigen binding). In addition, the capture platform design could be utilized for multidetection capability. It is important to note, however, that quantification of unfunctionalized dendrimers would require a labeling strategy for this capture assay approach to be used. 

**Figure 4:**
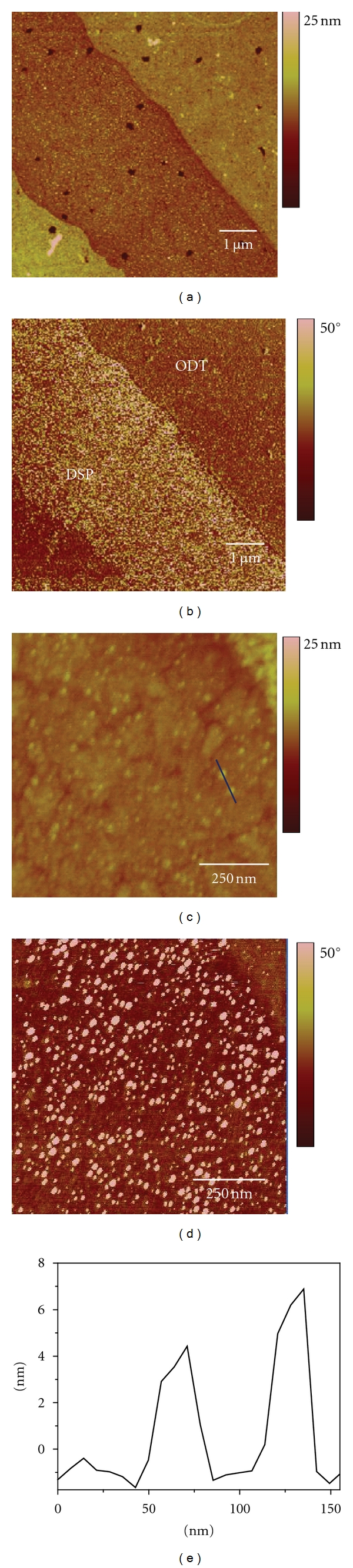
SPM image of capture surface patterning: (a) height and (b) phase (8 × 8 *μ*m), zoomed in analysis; (c) height and (b) phase of capture stripe (avidin immobilized on DSP) containing bound G4 biotinylated PAMAM dendrimers. Cross-section analysis of bound G4 biotinylated PAMAM dendrimers in (d) is shown in (e). The capture platform was exposed to 200 *μ*L of 5.0 *μ*mol/L concentration of G4 biotinylated PAMAM dendrimer solution.

**Figure 5:**
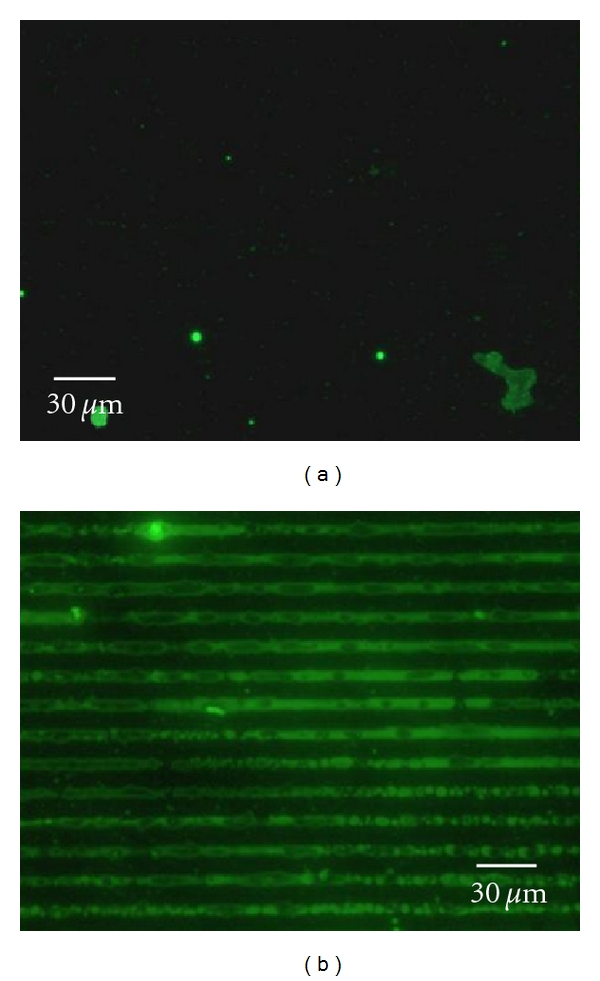
Fluorescence image of capture surface assay utilizing Alexa Fluor conjugated avidin: (a) blank and (b) G4 biotinylated PAMAM dendrimers bound to avidin capture substrate.

The capture assay platform also has the ability to selectively pattern PAMAM dendrimers onto a surface, as shown in Figure 4. In this study a PDMS stamp with a positive structure of 10 *μ*m wide stripes separated by 5 *μ*m stripes was used to pattern G4 biotinylated PAMAM dendrimers using the assay approach. Figures 4(a) and 4(b) show the height and phase analysis of the patterned surface. The bright stripes in the height analysis (Figure 4(a)) correspond to the ODT layer (10 *μ*m wide), and the darker stripe corresponds to the DSP layer (5 *μ*m wide). The height difference (~2.5 nm) is consistent with the differing dimensions of ODT and DSP monolayers. It can be seen from the phase analysis that the captured dendrimers are more easily identifiable in the DSP stripe (Figure 4(b)). Figures 4(c) and 4(d) present higher-resolution height and phase analysis of the patterned G4 biotinylated PAMAM dendrimers. In these SPM images the individual dendrimers bound to the DSP stripe can be ascertained. The dendrimers are individually distributed within the DSP stripe. The dendrimers do not clump together and multilayers of dendrimers are not evident. Furthermore, the ODT layers do not show any evidence of nonspecific adsorption. Cross-sectional height profiles (~4.5 nm) are consistent with G4 dendrimers. The slight variabilities in the height may be due to variations in how the biotin on the dendrimer surface is bound to the avidin capture platform. Further validation of the patterning of the G4 biotinylated PAMAM dendrimers was conducted by tagging the bound dendrimers with Alexa Fluor conjugated avidin, as shown in Figure 5. Fluorescence imaging verifies that the dendrimers are patterned on the avidin capture surface over large surface areas. Analysis of the blank confirms that nonspecific adsorption of G4 biotinylated PAMAM dendrimers is minimal. The bound patterned dendrimers have been found to be stable on the capture platform for >1 year under ambient storage conditions. Although, methods such as direct microcontact printing [42] dip-pen lithography [43], and electron-beam patterning [44] of dendrimers have been reported previously, they do not provide the selective patterning of this approach. The dimensions and profiles of the patterned dendrimers can be modified using different PDMS platforms. 

## 4. Conclusions

This paper has demonstrated the potential value of immunoimaging SPM to detect and quantify G4 biotinylated PAMAM dendrimers. Results were presented that showed that this new approach using an immunoassay platform provides a 1000-fold improvement in the limit of detection of dendrimers compared to current quantification methods using HPLC. The range of dendrimers that could be evaluated with this approach is potentially limited to the availability of analyte capture and labeling strategies. Findings have also shown the potential of this platform to pattern dendrimers for sensor and nanodevice applications.
